# Ethnic and Adipose Depot Specific Associations Between DNA Methylation and Metabolic Risk

**DOI:** 10.3389/fgene.2020.00967

**Published:** 2020-09-29

**Authors:** Carmen Pheiffer, Tarryn Willmer, Stephanie Dias, Yoonus Abrahams, Johan Louw, Julia H. Goedecke

**Affiliations:** ^1^Biomedical Research and Innovation Platform, South African Medical Research Council, Cape Town, South Africa; ^2^Division of Medical Physiology, Faculty of Medicine and Health Sciences, University of Stellenbosch, Stellenbosch, South Africa; ^3^Non-Communicable Diseases Research Unit, South African Medical Research Council, Cape Town, South Africa; ^4^Division of Exercise Science and Sports Medicine, Department of Human Biology, University of Cape Town, Cape Town, South Africa

**Keywords:** DNA methylation, ethnicity, obesity, adipose tissue, gluteal, abdominal

## Abstract

**Background:**

Metabolic risk varies according to body mass index (BMI), body fat distribution and ethnicity. In recent years, epigenetics, which reflect gene-environment interactions have attracted considerable interest as mechanisms that may mediate differences in metabolic risk. The aim of this study was to investigate DNA methylation differences in abdominal and gluteal subcutaneous adipose tissues of normal-weight and obese black and white South African women.

**Methods:**

Body composition was assessed using dual-energy x-ray absorptiometry and computerized tomography, and insulin sensitivity was measured using a frequently sampled intravenous glucose tolerance test in 54 normal-weight (BMI 18–25 kg/m^2^) and obese (BMI ≥ 30 kg/m^2^) women. Global and insulin receptor (*INSR*) DNA methylation was quantified in abdominal (ASAT) and gluteal (GSAT) subcutaneous adipose depots, using the Imprint methylation enzyme-linked immunosorbent assay and pyrosequencing. *INSR* gene expression was measured using quantitative real-time PCR.

**Results:**

Global DNA methylation in GSAT varied according to BMI and ethnicity, with higher levels observed in normal-weight white compared to normal-weight black (*p* = 0.030) and obese white (*p* = 0.012) women. Pyrosequencing of 14 CpG sites within the *INSR* promoter also showed BMI, adipose depot and ethnic differences, although inter-individual variability prevented attainment of statistical significance. Both global and *INSR* methylation were correlated with body fat distribution, insulin resistance and systemic inflammation, which were dependent on ethnicity and the adipose depot. Adipose depot and ethnic differences in *INSR* gene expression were observed.

**Conclusion:**

We show small, but significant global and *INSR* promoter DNA methylation differences in GSAT and ASAT of normal-weight and obese black and white South African women. DNA methylation in ASAT was associated with centralization of body fat in white women, whereas in black women DNA methylation in GSAT was associated with insulin resistance and systemic inflammation. Our findings suggest that GSAT rather than ASAT may be a determinant of metabolic risk in black women and provide novel evidence that altered DNA methylation within adipose depots may contribute to ethnic differences in body fat distribution and cardiometabolic risk.

## Introduction

Body fat distribution rather than fat mass is increasingly being recognized for its role in metabolic risk (Preis et al., 2010; Shay et al., 2011). Increased visceral adipose tissue (VAT) is associated with obesity-related complications such as type 2 diabetes, while subcutaneous adipose tissue (SAT) is considered relatively benign (Kwon et al., 2017). However, inter-depot variability between SAT depots exists. The accumulation of abdominal SAT (ASAT) has been linked to insulin resistance and obesity (Goodpaster et al., 1997), whereas gluteal SAT (GSAT) is less affected by these metabolic complications, and may even be considered protective (Manolopoulos et al., 2010). Studies in the United States (Lovejoy et al., 1996) and South Africa (Goedecke et al., 2009b) have shown ethnic variation in body fat distribution and metabolic risk. Black African women are more insulin resistant than women of European descent (white), despite having a more favorable body fat distribution, characterized by less VAT and more SAT, in particular gluteo-femoral SAT. Previously, we ascribed this paradox to decreased adipogenic capacity (Goedecke et al., 2011) and increased inflammation (Evans et al., 2011) in GSAT of Black African women, although the mechanisms underlying these differences are not yet fully elucidated.

DNA methylation is a key epigenetic mechanism that may mediate physiological and functional differences between ASAT and GSAT depots, and ethnic differences in body fat distribution and insulin resistance (Arner et al., 2016). The process involves the addition of a methyl group to the fifth carbon position of cytosine residues within cytosine-phosphate-guanine (CpG) dinucleotides, primarily in promoter regions (Bird, 2002), although non-promoter methylation has been suggested to also play a role in disease (Jones, 2012; Pheiffer et al., 2016). DNA methylation is associated with chromatin modifications and gene silencing, although the molecular mechanisms whereby DNA methylation modulates gene expression are not fully known (Zhu et al., 2003). Previously, we reported aberrant global DNA methylation, a crude estimate of overall genomic methylation associated with genomic and chromosomal instability, and gene or locus specific-DNA methylation during high-fat feeding and type 2 diabetes (Pheiffer et al., 2014, 2016; Matsha et al., 2016a, b). Others have reported differential methylation of several genes including insulin (Kuroda et al., 2009), glucose transporter 4 (Yokomori et al., 1999), adiponectin (Kim et al., 2015) and peroxisome proliferator-activated receptor gamma (PPARγ2) (Fujiki et al., 2009), which play an important role in glucose homeostasis. Recently, Ott et al. (2019) demonstrated that altered insulin receptor (*INSR*) promoter methylation in adipose tissue is associated with insulin resistance. The INSR is an important protein in the insulin signaling pathway and dysregulated expression has been demonstrated in muscle and adipocytes from obese, insulin-resistant people and patients with type 2 diabetes (Fröjdö et al., 2009). DNA methylation is reversible, therefore identification of aberrant DNA methylation patterns in genes critical to metabolic regulation may provide unique opportunities for intervention strategies. Lifestyle modifications such as diet (Obeid et al., 2018; Sae-Lee et al., 2018) and exercise (Rönn et al., 2013; Fabre et al., 2018) have been shown to reprogram altered DNA methylation, thus may lead to restoring metabolic homeostasis and preventing disease progression.

We hypothesized that altered DNA methylation profiles in ASAT and GSAT, through its regulatory effects on differential gene expression between these depots (Rantalainen et al., 2011; Karastergiou et al., 2013), contribute to ethnic differences in body fat distribution and metabolic risk of South African women. We profiled global and *INSR* promoter DNA methylation in ASAT and GSAT of normal-weight (BMI 18–25 kg/m^2^) and obese (BMI ≥ 30 kg/m^2^) black and white South African women. To further elucidate the role of DNA methylation, we sought to identify associations between DNA methylation, body fat distribution, insulin resistance and systemic inflammation.

## Materials and Methods

### Study Participants

The study population consisted of 14 normal-weight (BMI 18–25 kg/m^2^) and 15 obese (BMI ≥ 30 kg/m^2^), and 13 normal-weight and 12 obese black and white South African women, respectively, who were recruited from advertisements in local newspapers and from churches, community centers and universities as previously described (Goedecke et al., 2009b). Inclusion criteria were women between 21 and 45 years old, pre-menopausal, either of self-reported European or Black African (Xhosa) ancestry (both parents), neither pregnant nor lactating, and not taking medications for infectious or metabolic disorders. The study was approved by the Research Ethics Committee of the Faculty of Health Sciences of the University of Cape Town (REC REF: 052/2003). All procedures were conducted according to the Declaration of Helsinki and all women gave written, informed voluntary consent after the procedures had been fully explained in the language of their choice.

### Study Procedures

Demographic information was collected using a questionnaire that included information on asset index (14 items that reflect individual and household wealth), housing density (number of persons living per room in house), education (grades passed) and employment (unemployed, students, informal employment, employed) as previously described (Goedecke et al., 2009b). Based on this information, a socioeconomic (SES) score was calculated. Anthropometric measurements (weight, height and waist) and body composition was assessed using dual-energy-x-ray absorptiometry (DXA) (Discovery-W, Software version 4.40; Hologic Inc., Medford, United States) (Evans et al., 2011). Abdominal visceral and subcutaneous adipose tissue areas were measured using computerized tomography (Toshiba X-press Helical Scanner; Toshiba, Tokyo, Japan). After an overnight fast, venous blood was collected from all women for measurement of plasma glucose and serum insulin concentrations. Subsequently, women underwent a 75 g oral glucose tolerance test (OGTT) as recommended by the World Health Organization (2006). On another day, participants underwent an insulin-modified frequently sampled intravenous glucose tolerance test from which insulin sensitivity (S_I_) and acute insulin response to glucose (AIR_g_) were calculated (Goedecke et al., 2009a). Paired ASAT and GSAT biopsy specimens were obtained by mini-liposuction after a 4 h fast (Evans et al., 2011). Approximately 2 ml of fat was extracted from each depot, washed with saline three times, divided into aliquots, snap frozen in liquid nitrogen, and stored at −80°C until analysis.

### Biochemical Analysis

Plasma glucose levels were measured using the glucose oxidase method (YSI 2300 STAT PLUS; YSI Life Sciences, Yellow Springs, United States). Serum insulin concentrations were quantified using immunochemiluminometric assays (ADVIA Centaur, Bayer Diagnostics, Tarrytown, United States). Serum concentrations of leptin (Linco Research, St Charles, MO, United States), high molecular weight adiponectin (Linco Research) and high-sensitive C-reactive protein (CRP) (Immun Diagnostik AG, Bensheim, Germany) were all analyzed using commercially available enzyme-linked immunosorbent assay (ELISA) kits according to the manufacturer’s protocols. Homeostatic model assessment of insulin resistance (HOMA-IR) was calculated using fasting glucose and insulin levels [(glucose (mmol/L) × insulin (pmol/L))/22.5].

### DNA Methylation Analysis

Genomic DNA was extracted from 100 mg of GSAT and ASAT using the QIAamp Mini kit, according to the manufacturer’s instructions (Qiagen, Hilden, Germany). DNA concentration and purity were quantified using the Nanodrop spectrophotometer (Nanodrop Technologies, Wilmington, United States). Global DNA methylation was measured with the Imprint Methylated DNA Quantification kit, according to the manufacturer’s instructions (Sigma-Aldrich, St. Louis, United States) as previously reported (Pheiffer et al., 2014). Briefly, 50 ng of genomic DNA was allowed to bind to the ELISA plate, in duplicate, where after the methylated fraction of DNA was detected using a 5-methylcytosine monoclonal antibody and quantified by an ELISA-like reaction. The absorbance was measured at 450 nm on a BioTek^®^ ELX 800 plate reader (BioTek Instruments Inc., Winooski, United States) and DNA methylation was quantified relative to the positive control included in the Imprint Methylated DNA Quantification kit. Pyrosequencing was performed with assays (Table 1) purchased from EpigenDX (Worcester, United States). Bisulfite conversion was performed on 500 ng of DNA using the EpiTect Fast DNA Bisulfite Kit (Qiagen, Hilden, Germany) as recommended by the manufacturer. Thereafter, PCR was conducted using the Pyromark PCR kit and pyrosequencing was performed with the PyroMark Gold Q96 reagent kit and the PyroMark Q96 pyrosequencer (Qiagen, Hilden, Germany). Negative, bisulfite conversion and pyrosequencing controls as recommended by the manufacturer were included. Pyrosequencing assays were validated using different ratios of methylated:unmethylated bisulfite converted DNA (0, 10, 25, 50, 75, 90, and 100%) (Qiagen, Valencia, United States), from which standard curves were constructed to determine primer sensitivity (Supplementary Figure S1). The nomenclature is based on sequence identities from EpigenDX.

**TABLE 1:** *INSR* pyrosequencing assays.

Methylation assay	Chromosomal location^a^	bp from TSS	# CpGs^b^
ADS1698	Chr19: 7294488–7294657	−959 to −994	7
ADS1697	Chr19: 7294766–7294992	−638 to −674	7

*^*a*^According to University of California, Santa Cruz (UCSC) Genome browser GRC38/hg38 assembly. ^*b*^Number of CpGs analyzed. bp, base pair; chr, chromosome; TSS, transcription start site.*

### Gene Expression Analysis

Total RNA was extracted from GSAT and ASAT biopsies using the miRNeasy Kit (Qiagen, Hilden, Germany) according to the manufacturer’s instructions. RNA yield and purity were measured using the NanoDrop ND-1000 Spectrophotometer (NanoDrop Products, Wilmington, United States). RNA was reverse transcribed using the High Capacity cDNA Reverse Transcription Kit (Life Technologies, Carlsbad, CA, United States). Messenger RNA (mRNA) levels were quantified using Taqman Universal Master Mix and gene expression assays (Life Technologies): *INSR* (Hs00961557_m1), peptidylprolyl isomerase A (*PPIA*) (Hs04194521_s1), ribosomal protein P0 (*RPLP0*) (Hs99999902_m1), and 18S (Hs03003631_g1). Samples were run in duplicate on a QuantStudio 7 Flex Real-Time PCR System (Life Technologies) and *INSR* expression levels calculated using the relative quantification standard curve method. NormFinder algorithm (Andersen et al., 2004) identified *RPLP0* as the most stably expressed gene, thus *INSR* levels were normalized to *RPLP0* as an endogenous control.

### *In silico* Analysis

To explore the functional significance of the investigated CpG sites, *in silico* analysis was conducted to identify potential transcription factor binding sites that span these regions. The transcription factor prediction software ALIBABA (Grabe, 2000) was used to identify putative transcription factors, which were further investigated using chromatin immunoprecipitation sequencing (ChIP-Seq) data from the University of California, Santa Cruz (UCSC) Genome browser^1^ (human GRCh37/hg38 assembly, accessed January 2020) derived from the Encode Consortium (ENCODE) database.

### Statistical Analysis

Statistical analysis was performed using STATA version 14.0 (StataCorp, College Station, United States). Data in Table 2 are presented as the mean and standard deviation (SD) if normally distributed or the median and interquartile range (25th and 75th percentile) if not normally distributed. The Shapiro–Wilk test was used to test for normality. DNA methylation and gene expression data are represented as the mean and standard error of the mean (SEM). Linear or quantile regression was used to investigate ethnicity and BMI effects on group means or medians, respectively. If significant effects were identified, groups were compared using student *t*-tests or the Mann–Whitney test as appropriate. Multivariable quantile regression was conducted to adjust for age. Spearman correlation analyses were conducted to investigate the association between DNA methylation and participant characteristics. A *p*-value < 0.05 was considered statistically significant.

**TABLE 2:** Characteristics of women according to ethnicity and BMI.

Characteristics	Black women		White women		Regression β co-efficient and 95% CI		
	Normal-weight	Obese	Normal-weight	Obese	Ethnicity	BMI	Ethnicity x BMI
**N**	14	15	13	12	54	54	54
**Demographic**							
Age (years)	23.5 (21.0–25.0)	25.0 (22.0–32.0)	25.0 (23.0–27.0)	30.0 (24.5–39.0)	−1.0 (–7.6;5.6)	7.0 (0.1;13.9)	−6.0 (–15.4;3.4)
Height (m)	158.6 (6.3)^a^	157.1 (6.9)^b^	170.6 (6.5)^a^	166.1 (5.3)^b^	−12.0 (−16.9;−7.1)***	−4.5 (−9.6;0.6)	3.1 (−3.9;10.0)
SES score	12.1 (9.6–14.0)^a^	11.1 (7.7–13.3)^b^	22.6 (21.1–24.3)^a^	23.2 (22.0–23.8)^b^	−11.0 (−14.0;−8.0)***	0.57 (−2.6;3.7)	−1.0 (−5.3;3.3)
**Adiposity/Body fat distribution**							
Body mass index (kg/m^2^)	23.2 (21.8–23.8)^a^	38.4 (35.1–41.6)^a^	22.6 (21.2–23.6)^b^	35.3 (32.1–41.5)^b^	0.3 (−3.7;4.4)	13.1 (8.8;17.3)***	2.4 (−3.4;8.2)
Body fat (%)	29.0 (26.5;32.7)^a^	47.3 (44.4-49.6)^a^	30.8 (25.5;35.1)^b^	45.6 (42.1;47.6)^b^	−1.5 (−6.5;3.5)	14.6 (9.4;19.8)***	3.4 (−3.7;10.5)
Weight (kg)	58.3 (54.6–62.7)^a^	98.3 (89.9–101.1)^a^	66.6 (64.9–68.2)^b^	94.3 (86.7–117.2)^b^	−8.4 (−18.3;1.5)	31.6 (21.3;41.9)***	8.5 (−5.6;22.6)
Waist circumference (cm)	76.3 (74.0–79.5)^a^	114.0 (102.6–19.1)^a^	81.6 (77.2–83.0)^b^	110.8 (100.0–15.5)^b^	−5.0 (−13.8;3.8)	28.4 (19.2;37.6)***	9.0 (−3.5;21.5)
Total SAT (cm^2^)	148.3 (134.7–215.0)^a^	614.5 (566.0–627.7)^a,c^	180.9 (136.9–243.0)^b^	494.1 (454.9–550.6)^b,c^	−32.6 (−96.1;30.9)	317.2 (249.2;385.3)***	148.9 (55.8;242.0)**
SSAT area (cm^2^)	86.7 (75.4–118.1)^a^	300.6 (277.1–351.5)^a,c^	110.9 (75.8–114.5)^b^	253.6 (213.5–294.0)^b,c^	−24.3 (−67.2;18.7)	139.5 (93.4;185.6)***	74.4 (11.3;137.4)*
VAT (cm^2^)	55.1 (42.6-65.0)^d^	107.6 (80.3–117.8)^d^	51.2 (46.2–77.4)^e^	153.2 (90.7–203.4)^e^	4.0 (−31.1;39.1)	78.2 (40.5;115.8)***	−25.7 (−77.1;25.8)
VAT/SAT	0.4 (0.02)^a^	0.2 (0.02)^a,d^	0.34 (0.03)	0.29 (0.04)^d^	0.1(−0.1;0.1)	−0.1 (−0.1;0.1)	−0.1 (−0.3;0.1)*
GSAT fat cell size (cm^2^)	4783.3 (4042.9–5347.6)^d^	6312.4 (5663.7–6590.1)^d^	4319.6 (3992.1–4837.1)^e^	5601.9 (5009.9–6453.4)^e^	598.9 (−279.7;1477.5)	1535.6 (622.4;2448.7)**	32.2 (−1223.7;1288.0)
Central fat mass (kg)	6.4 (5.8–8.6)^a^	23.6 (19.8–24.7)^a^	8.9 (5.7–10.5)^b^	21.8 (18.0–26.6)^b^	−2.1 (−6.7;2.5)	13.7 (9.0;18.4)***	3.5 (−2.9;9.9)
Leg fat mass (kg)	7.7 (6.7–9.5)^a^	16.2 (13.4–18.8)^a^	8.8 (6.9–10.2)^b^	16.8 (16.0–20.4)^b^	−1.0 (−4.3;2.4)	8.2 (4.8;11.6)***	0.2 (−4.4;4.9)
Central fat mass%^γ^	42.8 (5.4)^a^	51.5 (3.5)^a^	44.7 (4.7)^d^	49.3 (3.7)^d^	−1.8 (−5.4;1.7)	4.6 (1.0; 8.2)*	4.1 (−0.8;9.0)
Leg fat mass%^γ^	46.9 (6.3)^a^	36.9 (4.2)^a^	44.8 (5.3)^d^	39.3 (4.4)^d^	2.1 (−2.0;6.2)	−5.5 (−9.7;−1.3)*	−4.5 (−10.2;1.2)
Central fat mass/Leg fat mass	0.9 (0.3)^a^	1.4 (0.3)^a^	1.0 (0.2)^d^	1.3 (0.2)^d^	−0.1 (−0.3;0.1)	0.3 (0.1;0.5)*	0.2 (−0.1;0.5)
**Metabolic parameters**							
Fasting Glucose (mmol/L)	4.4 (4.1–4.6)	4.5 (4.4–4.7)	4.2 (4.2–4.5)^c^	4.6 (4.3–4.7)^c^	0.2 (–0.1;0.5)	0.4 (0.1;0.7)*	–0.28 (−0.7; 0.2)
2h-Glucose (mmol/L)	4.9 (1.2)^c,d^	5.6 (1.0)^c,e^	3.7 (0.7)^a,d^	4.7 (0.6)^a,e^	1.2 (0.5;1.9)**	1.0 (0.3;1.8)**	–0.3 (−1.3;0.7)
Fasting Insulin (pmol/L)	8.5 (4.8–12.5)^c,d^	15.9 (9.8–21.0)^c,e^	4.5 (3.0–5.1)^c,d^	8.7 (5.5–11.7)^c,e^	4.0 (–1.5;9.5)	4.7 (–1.1;10.4)	2.7 (−5.1;10.5)
HOMA-IR	1.6 (0.9–2.0)^c^	3.2 (1.9–4.5)^c^	0.8 (0.7–0.9)^d^	1.7 (0.9–2.4)^d^	0.9 (–0.2;1.9)	1.2 (0.1;2.3)*	0.4 (−1.1;1.8)
S_I_ (x10^–4^ min^–1^/[uU/ml]	1.9 (1.2–3.3)^d^	1.1 (0.7–2.3)^c^	6.3 (4.6–7.4)^d,e^	3.5 (1.5–4.7)^d,e^	–3.7 (–5.6;–1.9)***	–2.7 (–4.7;–0.7)*	1.4 (−1.3;4.0)
AIRg (pmol/l)	159.9 (117.2–252.7)^a^	254.2 (176.1–337.5)^b^	48.2 (31.2–81.8)^a^	68.2 (46.2–73.4)^b^	113.2 (34.4;192.1)**	19.6 (–64.0;103.3)	72.7 (−39.2;184.7)
Adiponectin (μg/ml)	6.7 (2.9)^d^	3.7 (2.1)^d^	6.7 (2.8)^e^	4.0 (2.2)^e^	0.1 (–2.0;1.9)	–2.7 (–4.8;−0.7)*	−0.2 (−3.0;2.6)
Leptin (ng/ml)	14.9 (7.8–17.9)^a^	63.0 (49.7–66.7)^a^	15.5 (9.8-32.2)^b^	52.4 (40.3–60.2)^b^	–0.9 (–14.7;12.9)	36.3 (21.9;50.7)***	12.1 (−7.5;31.8)
C-reactive protein (μg/ml)	1.0 (1.8–4.7)^d^	3.7 (7.9–9.2)^d^	0.6 (0.8–1.9)^a^	2.9 (7.5–8.7)^a^	1.5 (–1.6;4.7)	6.3 (3.0;9.5)***	−0.8 (−5.2;3.7)

Data expressed as the median (25th–75th percentile) or mean (SD) for normally distributed variables. ^γ^Calculated as a percentage of total body fat. Linear or quantile regression was used to investigate ethnicity and BMI effects on group means or medians, respectively. β co-efficient and 95% CI is indicated. *p < 0.05, **p < 0.01, ***p < 0.001 indicated in bold. Superscripts with similar letters represent significant differences between means and medians, ^*c*^*p* ≤ 0.05, ^*d,e*^*p* < 0.01, ^*a,b*^*p* < 0.001. AIR_*g*_, acute insulin response to glucose; 95% CI, 95% confidence interval; GSAT, gluteal subcutaneous adipose tissue; HOMA-IR, Homeostasis model for insulin resistance; SAT, subcutaneous adipose tissue; SES score, socioeconomic score; S_*I*_, insulin sensitivity index; SSAT: superficial subcutaneous adipose tissue VAT/SAT: Ratio of visceral adipose tissue to SAT.

## Results

### Participant Characteristics

Participant characteristics are presented in Table 2. As designed, obese women had higher BMIs and greater body fat measures than their normal-weight counterparts. Although BMI and total body fat did not differ by ethnicity, obese black women had more total SAT (*p* = 0.03 and *p* = 0.002 for ethnicity × BMI interaction) and lower VAT/SAT (*p* = 0.004 and *p* = 0.02 for ethnicity × BMI interaction) than obese white women. Fasting glucose concentrations were not different between the groups, but 2-h glucose and fasting insulin concentrations were higher in normal-weight black compared to normal-weight white women, and in obese white women compared to their normal-weight counterparts. HOMA-IR was higher and insulin sensitivity was lower with a correspondingly higher acute insulin response to glucose in black compared to white women. Insulin sensitivity was also lower in obese women compared to their normal-weight counterparts. Adiponectin concentrations were lower, while leptin and CRP concentrations were higher in obese compared to normal-weight women, irrespective of ethnicity, while SES was lower in black compared to white women, irrespective of BMI.

### DNA Methylation Differences Across Ethnicity and Body Mass Index

**FIGURE 1:**
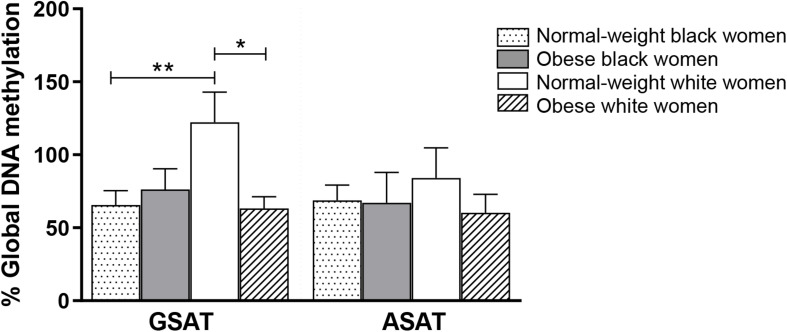
Global DNA methylation in gluteal (GSAT) and abdominal (ASAT) subcutaneous adipose tissue of normal-weight and obese black and white South African women. Data are presented as the mean and SEM (*n* = 11–15 per group). *p < 0.05, **p < 0.01 adjusted for age using quantile regression.

**FIGURE 2:**
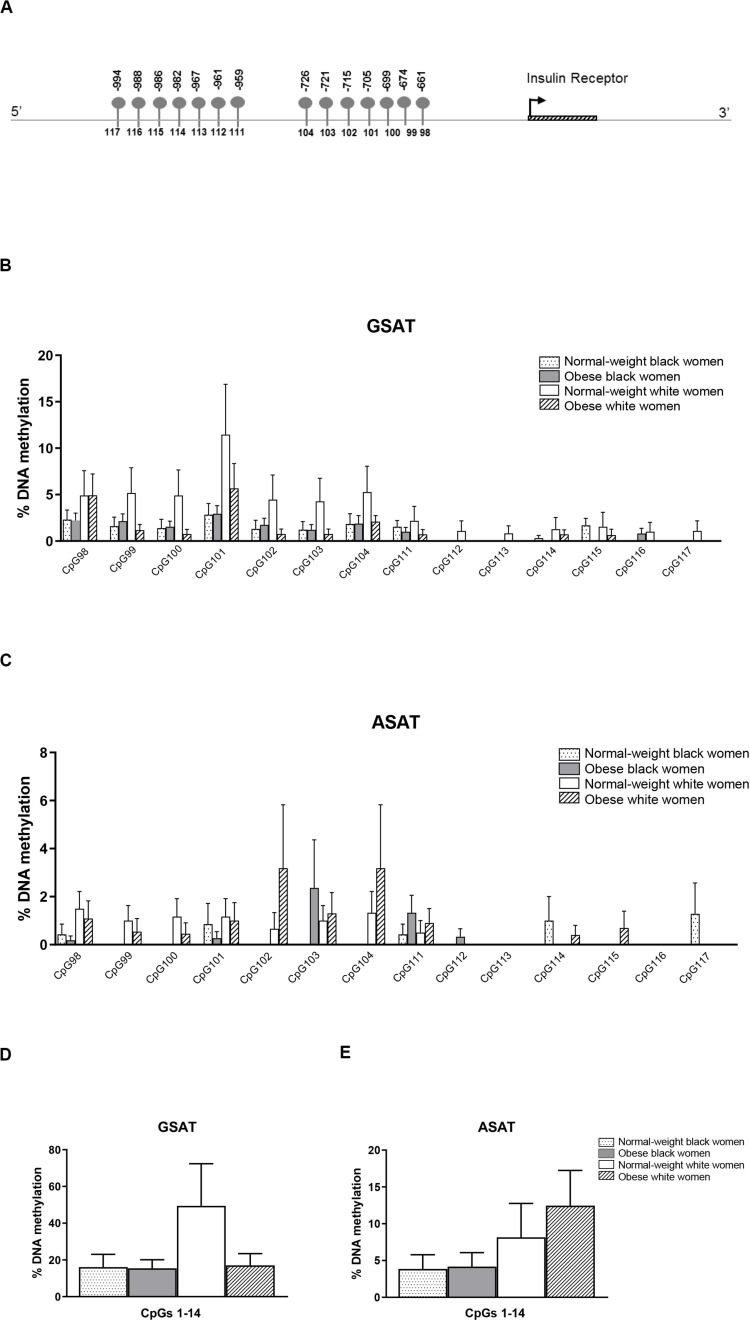
Pyrosequencing of 14 CpG sites across the *INSR* promoter in gluteal (GSAT) and abdominal (ASAT) adipose tissue. **(A)** Schematic diagram of the *INSR* gene illustrating CpG sites in the promoter region that were analyzed. The distance between the CpG sites and the transcription start site is indicated. CpG specific methylation levels in GSAT **(B)** and ASAT **(C)** of normal-weight and obese black and white women. Cumulative DNA methylation levels across all 14 CpG sites in GSAT **(D)** and ASAT **(E)**. Data are presented as the mean and SEM (*n* = 8–11 per group).

Quantile regression showed a significant ethnicity x BMI interaction for global DNA methylation in GSAT (*p* = 0.024), such that higher levels were observed in normal-weight white compared to normal-weight black (122.20 ± 20.67 vs. 65.51 ± 9.96, *p* = 0.003) or obese white (122.20 ± 20.67 vs. 63.17 ± 8.03, *p* = 0.011) women (Figure 1). No significant difference in global DNA methylation levels in GSAT were observed between normal-weight and obese black women. Furthermore, no differences in global DNA methylation were observed in ASAT. Pyrosequencing of the *INSR* (Figure 2A) showed significant inter-individual DNA methylation variation across the 14 CpG sites analyzed with no trends nor statistically significant differences observed in GSAT (Figure 2B) nor ASAT (Figure 2C). Similarly, no difference in cumulative methylation was observed in GSAT (Figure 2D) or ASAT (Figure 2E).

### Depot-Specific Differences

**FIGURE 3:**
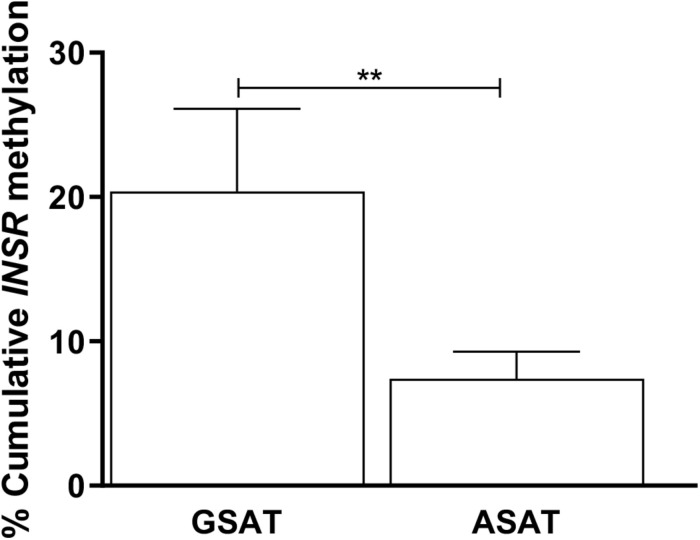
Paired analysis of cumulative *INSR* promoter methylation in gluteal (GSAT) and abdominal (ASAT) subcutaneous adipose tissue. Data are presented as the mean and SEM (*n* = 35 per group). **p < 0.01.

Matched samples of GSAT and ASAT were analyzed to investigate depot-specific differences. A trend toward lower percentage global DNA methylation in ASAT compared to GSAT was observed (69.89 ± 9.01 vs. 86.44 ± 9.57, *p* = 0.060), although the difference was not statistically significant (data not shown). *INSR* DNA methylation was significantly lower in ASAT compared to GSAT (7.40 ± 1.88 vs. 20.40 ± 5.72, *p* = 0.004) (Figure 3). Quantile regression for repeated measures showed that lower *INSR* methylation in ASAT compared to GSAT remained significant after adjusting for age, BMI and ethnicity (*p* = 0.001).

### Association Between DNA Methylation and Participant Characteristics

**FIGURE 4:**
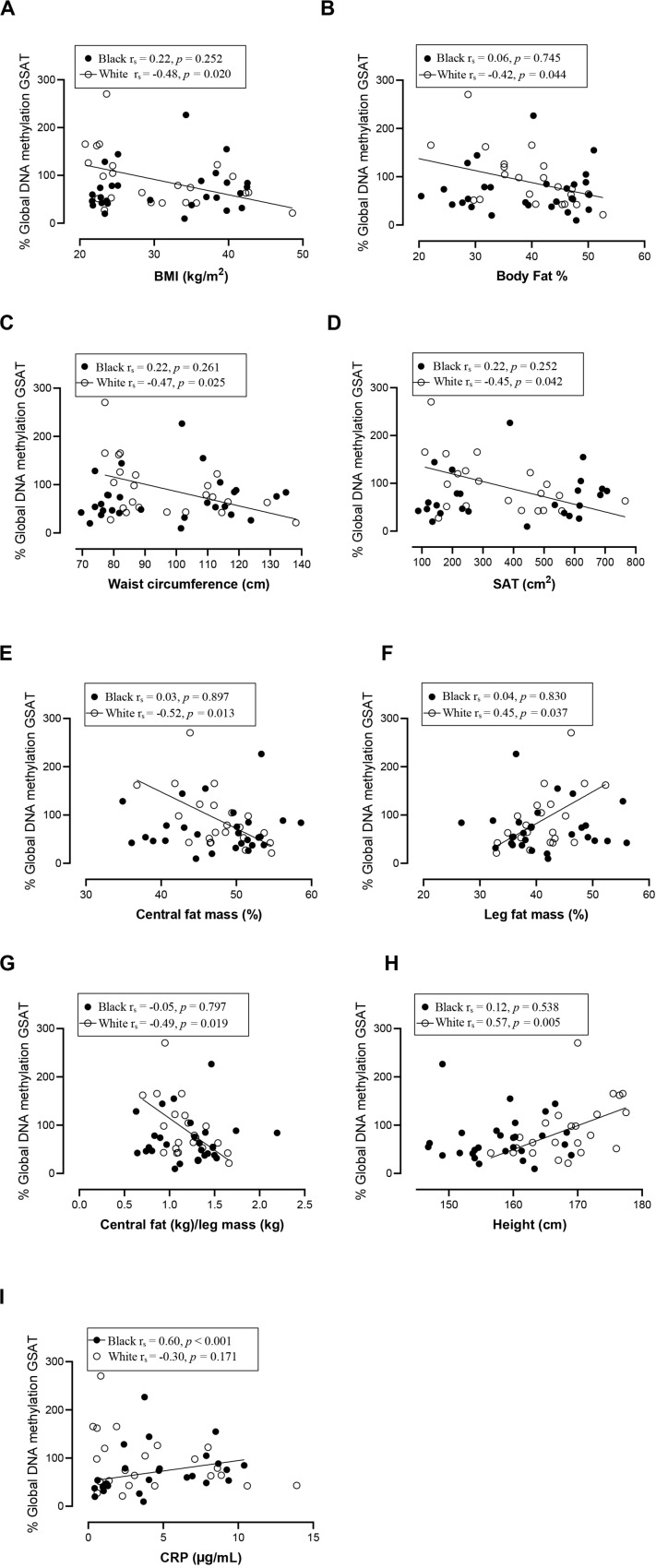
Relationship between global DNA methylation in GSAT and BMI **(A)**, Body fat percentage **(B)**, Waist circumference **(C)**, SAT **(D)**, Central fat mass percentage **(E)**, Leg fat mass percentage **(F)**, Ratio central to leg fat mass **(G)**, Height **(H),** and CRP **(I)** in black and white women. Each data point represents values for individual women. Rho (*r*_s_) and *p*-values were calculated using Spearman’s correlation test. To aid visualization regression lines are shown for significant associations. BMI, Body mass index; CRP, C-reactive protein; GSAT, gluteal adipose tissue; resistance; SAT, subcutaneous adipose tissue.

**FIGURE 5:**
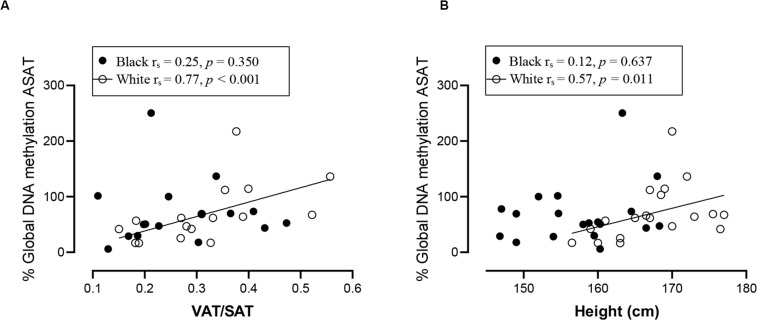
Relationship between global DNA methylation in ASAT and VAT/SAT ratio **(A)** and Height **(B)** in black and white women. Each data point represents values for individual women. Rho (*r*_s_) and *p*-values were calculated using Spearman’s correlation test. To aid visualization regression lines are shown for significant associations. ASAT, abdominal adipose tissue; VAT/SAT: Ratio of visceral to subcutaneous adipose tissue.

**FIGURE 6:**
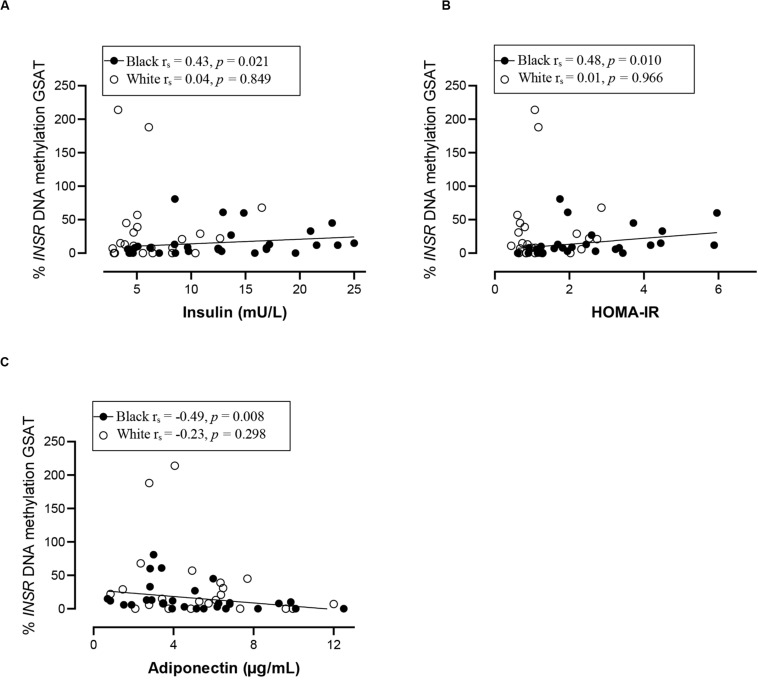
Relationship between *INSR* methylation in GSAT and Insulin concentrations **(A)** HOMA-IR **(B)** and Adiponectin concentrations **(C)** in black and white women. Each data point represents values for individual women. Rho (*r*_s_) and *p*-values were calculated using Spearman’s correlation test. To aid visualization regression lines are shown for significant associations. GSAT, gluteal adipose tissue; HOMA-IR, Homeostatic model assessment-insulin resistance; VAT/SAT: Ratio of visceral to subcutaneous adipose tissue.

Spearman correlation analysis was conducted to investigate the association between DNA methylation, body fat distribution, insulin resistance and systemic inflammation separately for ethnic group (Figures 4–6). Since the data was not normally distributed, regression lines are shown for significant associations to aid visualization. In white women only, global DNA in GSAT was negatively correlated with markers of total (BMI and body fat percentage) and centralization (waist, SAT and central fat as a percentage of total body fat) of body fat, and positively correlated with lower-body fat (leg fat mass as a percentage of total body fat) (Figure 4). Global DNA in ASAT correlated with VAT/SAT (Figure 5). In black women only, global DNA methylation in GSAT was correlated with C-reactive protein (Figure 4). Intriguingly, global DNA methylation levels in both GSAT and ASAT were positively correlated with height in white, but not in black women (Figures 4, 5). The associations between global DNA methylation in GSAT and height (*p* = 0.038), central fat as a percentage of total body fat (*p* = 0.027), ratio of central to leg fat (*p* = 0.021) and global DNA methylation in ASAT and VAT/SAT (*p* = 0.025) remained statistically significant, with borderline significance for CRP (*p* = 0.067) after adjusting for age, possibly due to the small sample size. Small, but significant positive correlations between cumulative methylation of 14 CpGs in the *INSR* gene in GSAT and fasting insulin concentrations (*p* = 0.021) and HOMA-IR (*p* = 0.021), and a negative correlation with adiponectin concentrations (*p* = 0.008) was observed in black, but not white women (Figure 6). These correlations did not withstand adjustment using quantile regression.

### *INSR* Gene Expression

**FIGURE 7:**
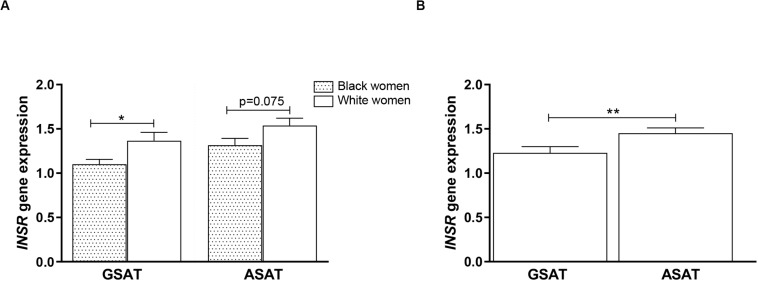
*INSR* gene expression in gluteal (GSAT) and abdominal (ASAT) subcutaneous adipose tissue of normal-weight and obese black and white South African women. **(A)** *INSR* expression according to ethnicity, irrespective of BMI status (*n* = 18–28 per group). **(B)** *INSR* expression in paired GSAT and ASAT samples, irrespective of ethnicity and BMI (*n* = 35 per group). *INSR* expression was normalized to the endogenous control *RPLP0* and data are presented as the mean and SEM. *p < 0.05, **p < 0.01.

To examine whether DNA methylation levels correlated with gene expression, *INSR* mRNA was measured in GSAT and ASAT using quantitative real-time PCR. *INSR* expression in GSAT and ASAT was ∼15% (*p* < 0.05) and ∼20% (*p* = 0.075) higher, respectively, in white compared to black women (Figure 7A). *INSR* expression did not differ significantly according to BMI. When comparing *INSR* gene expression between depots, median *INSR* gene expression was ∼20% higher in ASAT compared to GSAT (*p* < 0.5) in all women (Figure 7B).

### Identification of Potential Transcription Factor Binding Sites

**FIGURE 8:**
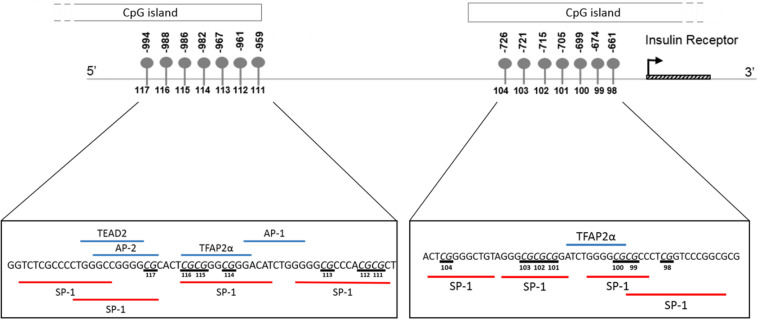
Schematic illustration showing *in silico* predicted transcription factors to the region of the *INSR* promoter investigated by pyrosequencing. Transcription factor binding sites within the region –1015 to –956 and –677 to –626 upstream of the *INSR* gene transcription start site were identified using ALIBABA 2.1 software. The following factors were identified: SP-1, Specificity Protein 1; TFAP2α, Transcription factor activating enhancer binding protein 2 alpha; AP-1, Activating protein 1; AP-2, Activator protein 2; TEAD2, TEA domain transcription factor 2. Underlined nucleotides indicate CpG sites.

*In silico* transcription factor binding site prediction within the investigated regions found several binding sites for Specificity protein 1 (SP-1), along with two for Transcription factor AP2 alpha (TFAP2α) and one each for TEA domain transcription factor 2 (TEAD2), Activating protein 1 (AP-1) and Activator protein 2 (AP-2) (Figure 8). Furthermore, the UCSC genome browser contained ChIP-Seq data for several transcription factors, such as SP-1, Upstream stimulatory factor 1 and 2 (USF1/2), Nuclear respiratory factor 1 (NRF1) and Ying-yang 1 (YY1) important for adipocyte differentiation, metabolism and insulin production within the wider CpG islands that spanned these regions (Supplementary Table S1).

## Discussion

The mechanisms that underlie BMI, body fat distribution and ethnic differences in metabolic risk are not yet fully elucidated. Increasing evidence implicates DNA methylation as a key epigenetic mechanism that may mediate physiological and functional differences between ASAT and GSAT depots, and ethnic differences in body fat distribution and insulin resistance. The present study measured global and *INSR* promoter DNA methylation in ASAT and GSAT of normal-weight and obese black and white South African women and provides novel evidence that altered DNA methylation within adipose depots may contribute to ethnic differences in body fat distribution and cardiometabolic risk. The main findings are (1) differences in global and *INSR* methylation according to BMI, adipose depot and ethnicity (2) hypermethylation of the *INSR* promoter in GSAT compared to ASAT and (3) ethnic- and adipose-depot specific associations between DNA methylation and body fat distribution, insulin sensitivity and systemic inflammation.

In our study global DNA methylation in GSAT of normal-weight white women was almost two-fold higher than that in both normal-weight black and obese white women. Global hypomethylation is associated with genomic and chromosomal instability and is widely reported during cancer, aging and metabolic disease (Toraño et al., 2012; Luttmer et al., 2013; Piyathilake et al., 2013; Unnikrishnan et al., 2018). Evidence for ethnic differences in global DNA methylation has been reported. Quantifying methylation of long interspersed repeat sequences (LINE-1), as a marker of global DNA methylation, Zhang et al. (2011) reported lower levels of methylation in the peripheral blood of black compared to white individuals in the United States, which is consistent with our findings in GSAT. In white women only, global DNA methylation in GSAT correlated with lower total and central subcutaneous fat mass and negatively with lower-body fat mass, whereas in ASAT global DNA was associated with increased VAT/SAT, a marker for cardiometabolic risk. Similar findings were reported by Keller et al. (2014) who quantified global DNA methylation in ASAT from a European population using the luminometric methylation assay and reported a positive association with waist circumference, a marker of metabolic risk. These findings suggest that in white women, global DNA methylation in GSAT may be protective, i.e., associated with lower adiposity, whereas in ASAT global DNA methylation is associated with cardiometabolic risk, i.e., higher VAT/SAT. These results are consistent with the hypothesis that GSAT is protective, whereas ASAT is associated with metabolic risk (Manolopoulos et al., 2010) and provides epigenetic evidence for the depot-specific associations with metabolic risk.

In contrast to white women, DNA methylation in GSAT rather than ASAT was associated with cardiometabolic risk in black women. In GSAT, global DNA methylation was correlated with higher systemic inflammation and *INSR* methylation was associated with insulin resistance in black women. In a previous study, black women were reported to have a higher inflammatory profile in GSAT compared to white women (Evans et al., 2011), suggesting that these cytokines may stimulate CRP release from the liver and increase cardiometabolic risk (Berg and Scherer, 2005). However, although CRP levels were higher in black compared to white women in our study, the difference was not statistically significant, possibly due to the smaller sample size. Notably, cumulative methylation across 14 CpGs of *INSR* promoter in GSAT was associated with higher insulin resistance (insulin concentrations and HOMA-IR) and lower adiponectin concentrations in black women. These findings suggest that DNA methylation may lead to altered expression of genes involved in the regulation of fat distribution, insulin sensitivity and inflammation, and in so doing partly mediate ethnic differences in adipose distribution and metabolic risk between black and white women. Due to the cross-sectional nature of the study, our findings are purely correlative and cannot be used to determine causality. However, this data adds to increasing evidence that implicate DNA methylation as a risk factor in mediating the differences between adipose depots, ethnicity and metabolic risk. Notably, associations between global DNA methylation and metabolic parameters were previously reported in peripheral blood (Zhang et al., 2011; Piyathilake et al., 2013). Zhang et al. (2011) reported that LINE-1 methylation in peripheral blood is associated with lower BMI and waist circumference and positively correlated with the VAT/SAT ratio in a US population, while Piyathilake et al. (2013) reported that LINE-1 methylation in peripheral blood is correlated with lower BMI and waist circumference in a European population, as reported in GSAT of white women. Our findings show that associations between DNA methylation in adipose tissue and body fat composition and distribution may be reflected in blood, lending support to the potential of using blood as biomarkers of cardiometabolic risk.

Intriguingly, global DNA methylation in GSAT and ASAT were associated with height in white women, who were on average 12 cm taller than black women. Height may reflect nutrition and environmental exposures during childhood, which are known to differ according to ethnicity in South Africa (Said-Mohamed et al., 2015). Black children are more prone to stunting, defined as having a height-for-age index more than two standard deviations below the World Health Organization Child Growth Standard median (World Health Organization, 2007). Stunting is correlated with poorer socio-economic status and metabolic risk, as observed for black women in this study. The significance of the ethnic disparities and associations between global DNA methylation, height and metabolic risk require further investigation.

Overall, *INSR* methylation in GSAT was higher than in ASAT, which was accompanied by higher *INSR* gene expression in ASAT and is consistent with a previous study conducted in premenopausal women (Karastergiou et al., 2013). Karastergiou et al. (2013) reported higher expression of insulin signaling genes in ASAT compared to GSAT in an apparently healthy and predominantly Caucasian population. To further explore the differences in *INSR* promoter methylation and gene expression in GSAT and ASAT, *in silico* analysis was conducted to identify putative transcription factors that bind to the region. Altered DNA methylation in this region, through its effects on chromatin structure, may affect transcription factor binding and gene regulation (Zhu et al., 2016). ALIBABA software predicted transcription factors SP-1, AP-1, AP-2, TEAD2 and TFAP2α to bind across the investigated CpG sites and are likely to regulate gene expression. Ott et al. (2019) similarly reported that SP-1 and AP-2 bind across this region of the *INSR*. Unfortunately, ChIP-Seq data did not confirm peaks for these transcription factors across the investigated regions which may be due to the lack of visceral and subcutaneous adipose tissue ChIP-seq datasets in the ENCODE database. ChIP-Seq identified transcription factors for adipocyte differentiation, metabolism and insulin production within the wider CpG islands that spanned this region, supporting its potential role in mediating the BMI, adipose depot and ethnic differences observed in this study. The data available on the UCSC genome browser is an accumulation of ChIP-Seq data from several tissues and there is currently a knowledge gap on whether these transcription factors act within adipose tissue specifically. Further studies to investigate the relationship between these transcription factors, DNA methylation and *INSR* gene expression in adipose tissue, and their role in mediating metabolic risk are warranted. In addition, ChIP-seq peaks for several histone related factors within the CpG islands were identified, which alludes to the complexity of *INSR* regulation, and suggest that other CpG sites or epigenetic mechanisms may contribute to regulate *INSR* gene expression.

The strength of the study is that global and *INSR* promoter DNA methylation was profiled in two different adipose depots of a well-characterized sample of age-matched normal-weight and obese black and white South African women. To our knowledge, this is the first study to investigate the relationship between DNA methylation, metabolic risk, ethnicity and adipose depot in African women. Nonetheless, these results must be interpreted with caution in light of several limitations. Study participants were all South African women and therefore generalizability to other population groups, or males, is unknown. Furthermore, the sample size was small and substantial individual heterogeneity between women led to large variances in the data and the study being underpowered to detect statistical differences in *INSR* promoter methylation. The small sample size also limited our ability to adjust for confounding factors including diet, physical activity, smoking and alcohol consumption that are known to influence DNA methylation (Christensen and Marsit, 2011; Ling and Rönn, 2019). The combination of a small sample and possible sampling bias further limits the ability to draw significant inferences from this study and to extrapolate the results to the general population. However, given the unique nature of the sample, the present study provides valuable information on the interactions between DNA methylation, obesity, adipose depot and ethnicity. Our findings pave the way for larger confirmatory studies with sufficient power to further probe these associations. Ideally longitudinal studies are required to investigate transition across various categories of BMI. Another limitation of the study is its cross-sectional nature, which prevents the exploration of a causal relationship between DNA methylation and metabolic risk. Adipose tissue consists of various cell types including adipocytes, mesenchymal stem cells, preadipocytes, macrophages, neutrophils, lymphocytes and endothelial cells (Esteve Ràfols, 2014), thus it is possible that DNA methylation profiles of these cells may have contributed to the altered DNA methylation patterns observed. Experiments utilizing purified adipocytes isolated from fat tissue are required to examine whether adipocyte-specific DNA methylation patterns may contribute to the ethnic differences in body fat distribution and metabolic risk observed in this study. However, previous studies have mainly used adipose tissue rather than purified adipocytes, thus comparison of our data to these studies is appropriate. Technical limitations include the relatively small number of CpGs studied. Pyrosequencing, although a highly reproducible and accurate method to quantify DNA methylation, is limited by the number of CpGs it can analyze (<10) (55,56), thus other important methylation sites may have been missed. Finally, although higher *INSR* methylation in GSAT was accompanied by lower transcript levels in GSAT compared to ASAT, *INSR* expression correlated with methylation levels in GSAT of normal-weight women only. Correlations were not observed in ASAT and in obese women, irrespective of ethnicity. It is generally believed that DNA methylation is associated with gene silencing, however, recent findings suggest that DNA methylation is functionally complex and may regulate mechanisms beyond gene silencing (Bird, 2002; Pheiffer et al., 2016). Other epigenetic mechanisms such as histone modifications (Castellano-Castillo et al., 2019) and microRNAs (Klöting et al., 2009; Hilton et al., 2019) may also contribute to differences in obesity and adipose distribution and should be studied to fully understand the mechanisms that underlie ethnic differences in metabolic risk.

## Conclusion

We show small, but significant global and gene-specific DNA methylation differences in GSAT and ASAT of normal-weight and obese black and white African women. Both global and *INSR* methylation were correlated with body fat distribution, insulin resistance and systemic inflammation, which were dependent on ethnicity and adipose depot. DNA methylation in ASAT was associated with centralization of body fat in white women, whereas in black women DNA methylation in GSAT was associated with insulin resistance and systemic inflammation. Our findings suggest that GSAT rather than ASAT may be a determinant of risk in black women and provide novel evidence that altered DNA methylation within adipose depots may contribute to ethnic differences in body fat distribution and cardiometabolic risk.

## Data Availability Statement

The raw data supporting the conclusions of this article will be made available by the authors on reasonable request.

## Ethics Statement

The studies involving human participants were reviewed and approved by The Research Ethics Committee of the Faculty of Health Sciences of the University of Cape Town (REC REF: 052/2003) approved the protocol. The patients/participants provided their written informed consent to participate in this study.

## Author Contributions

CP conceptualized the study and methodology. JG was responsible for the initial study cohort design, subject recruitment, and sample and data collection. CP, TW, and SD conducted the experiments. YA performed the *in silico* analysis. CP wrote the original manuscript draft. All the authors reviewed and revised manuscript drafts and approved the final manuscript.

## Conflict of Interest

The authors declare that the research was conducted in the absence of any commercial or financial relationships that could be construed as a potential conflict of interest.

## Footnotes

1 https://genome.ucsc.edu/

## Supplementary Material

FIGURE S1 Validation of pyrosequencing assays.

TABLE S1 ChIP-seq data for proteins binding the CpG island region of the *INSR* promoter.
