# Supplementary material for: Ethnic and Adipose Depot Specific Associations Between DNA Methylation and Metabolic Risk
Source: Front Genet. 2020 Sep 29;11:967. doi: 10.3389/fgene.2020.00967 (PMC7550664; doi:10.3389/fgene.2020.00967)
Supplement: FIGURE S1 — Validation of pyrosequencing assays. [file Data_Sheet_1.docx]

**Ethnic and adipose depot specific associations between DNA methylation and metabolic risk**

**Carmen Pheiffer^1,2^*, Tarryn Willmer^1^, Stephanie Dias^1^, Yoonus Abrahams^1,2^, Julia H. Goedecke^3,4^**

^1^Biomedical Research and Innovation Platform, South African Medical Research Council, Tygerberg, Western Cape, South Africa

^2^Division of Medical Physiology, Faculty of Medicine and Health Sciences, University of Stellenbosch, Tygerberg, Western Cape, South Africa

^3^Non-Communicable Diseases Research Unit, South African Medical Research Council, Tygerberg, Western Cape, South Africa

^4^Division of Exercise Science and Sports Medicine, Department of Human Biology, University of Cape Town, Newlands, Western Cape, South Africa.

***Correspondence:**Dr Carmen Pheiffer

[carmen.pheiffer@mrc.ac.za](mailto:carmen.pheiffer@mrc.ac.za)


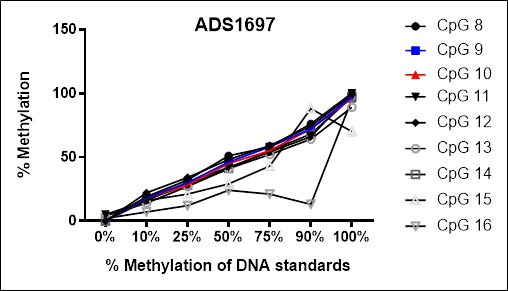


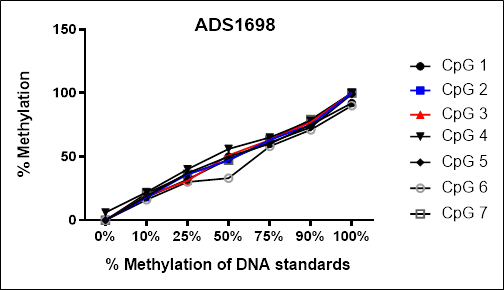


**Supplementary Figure 1.** Validation of pyrosequencing assays. The quality of assays was tested with methylated standards (0% to 100%). CpGs 15 and 16 were excluded from further analysis due to poor performance.

| CpG No. | EpigenDX Nomenclature |
| --- | --- |
| CpG 1 | 98 |
| CpG 2 | 99 |
| CpG 3 | 100 |
| CpG 4 | 101 |
| CpG 5 | 102 |
| CpG 6 | 103 |
| CpG 7 | 104 |
| CpG 8 | 111 |
| CpG 9 | 112 |
| CpG 10 | 113 |
| CpG 11 | 114 |
| CpG 12 | 115 |
| CpG 13 | 116 |
| CpG 14 | 117 |
