# Supplementary material for: Ethnic and Adipose Depot Specific Associations Between DNA Methylation and Metabolic Risk
Source: Front Genet. 2020 Sep 29;11:967. doi: 10.3389/fgene.2020.00967 (PMC7550664; doi:10.3389/fgene.2020.00967)
Supplement: TABLE S1 — ChIP-seq data for proteins binding the CpG island region of the INSR promoter. [file Table_1.docx]

**Supplementary Table 1** ChIP-seq data for proteins binding the CpG island region of the *INSR* promoter

| **Symbol** | | |  | **Description** |
| --- | --- | --- | --- | --- |
| AGO2 |  | Argonaute RISC Catalytic Component 2 | | |
| ARID3A |  | AT-Rich Interaction Domain 3A | | |
| ASH2L |  | ASH2 Like, Histone Lysine Methyltransferase Complex Subunit | | |
| ATF7 |  | Activating Transcription Factor 7 | | |
| BACH1 |  | BTB Domain and CNC Homolog 1 | | |
| BCL3 |  | BCL3 Transcription Coactivator | | |
| BHLHE40 |  | Basic Helix-Loop-Helix Family Member E40 | | |
| CHD1 |  | Chromodomain Helicase DNA Binding Protein 1 | | |
| CHD4 |  | Chromodomain Helicase DNA Binding Protein 4 | | |
| CREB1 |  | CAMP Responsive Element Binding Protein 1 | | |
| CREM |  | CAMP Responsive Element Modulator | | |
| CTCF |  | CCCTC-Binding Factor | | |
| DACH1 |  | Dachshund Family Transcription Factor 1 | | |
| DPF2 |  | Double PHD Fingers 2 | | |
| E2F6 |  | E2F Transcription Factor 6 | | |
| E2F8 |  | E2F Transcription Factor 8 | | |
| EBF1 |  | EBF Transcription Factor 1 | | |
| EED |  | Embryonic Ectoderm Development | | |
| EGR1 |  | Early Growth Response 1 | | |
| ELF1 |  | E74 Like ETS Transcription Factor 1 | | |
| EP300 |  | E1A Binding Protein P300 | | |
| ESRRA |  | Estrogen Related Receptor Alpha | | |
| ETS1 |  | ETS Proto-Oncogene 1, Transcription Factor | | |
| EZH2 |  | Enhancer of Zeste 2 Polycomb Repressive Complex 2 Subunit | | |
| FOSL2 |  | FOS Like 2, AP-1 Transcription Factor Subunit | | |
| FOXA1 |  | Forkhead Box A1 | | |
| FOXK2 |  | Forkhead Box K2 | | |
| FUS |  | FUS RNA Binding Protein | | |
| GABPA |  | GA Binding Protein Transcription Factor Subunit Alpha | | |
| GATAD2B |  | GATA Zinc Finger Domain Containing 2B | | |
| HCFC1 |  | Host Cell Factor C1 | | |
| HDAC1 |  | Histone Deacetylase 1 | | |
| HDAC2 |  | Histone Deacetylase 2 | | |
| HDAC6 |  | Histone Deacetylase 6 | | |
| HNF4A |  | Hepatocyte Nuclear Factor 4 Alpha | | |
| HNF4G |  | Hepatocyte Nuclear Factor 4 Gamma | | |
| HNRNPK |  | Heterogeneous Nuclear Ribonucleoprotein K | | |
| HNRNPL |  | Heterogeneous Nuclear Ribonucleoprotein L | | |
| HNRNPLL |  | Heterogeneous Nuclear Ribonucleoprotein L Like | | |
| IKZF1 |  | IKAROS Family Zinc Finger 1 | | |
| IKZF2 |  | IKAROS Family Zinc Finger 2 | | |
| IRF1 |  | Interferon Regulatory Factor 1 | | |
| IRF3 |  | Interferon Regulatory Factor 3 | | |
| JUND |  | JunD Proto-Oncogene, AP-1 Transcription Factor Subunit | | |
| KDM1A |  | Lysine Demethylase 1A | | |
| KDM4A |  | Lysine Demethylase 4A | | |
| KDM5A |  | Lysine Demethylase 5A | | |
| L3MBTL2 |  | L3MBTL Histone Methyl-Lysine Binding Protein 2 | | |
| MAX |  | MYC Associated Factor X | | |
| MEF2A |  | Myocyte Enhancer Factor 2A | | |
| MEF2B |  | Myocyte Enhancer Factor 2B | | |
| MEF2C |  | Myocyte Enhancer Factor 2C | | |
| MLLT1 |  | MLLT1 Super Elongation Complex Subunit | | |
| MNT |  | MAX Network Transcriptional Repressor | | |
| MTA3 |  | Metastasis Associated 1 Family Member 3 | | |
| MXI1 |  | MAX Interactor 1, Dimerization Protein | | |
| MYC |  | MYC Proto-Oncogene, BHLH Transcription Factor | | |
| NBN |  | Nibrin | | |
| NCOR1 |  | Nuclear Receptor Corepressor 1 | | |
| NEUROD1 |  | Neuronal Differentiation 1 | | |
| NFIC |  | Nuclear Factor I C | | |
| NR2F2 |  | Nuclear Receptor Subfamily 2 Group F Member 2 | | |
| NR2F6 |  | Nuclear Receptor Subfamily 2 Group F Member 6 | | |
| NRF1 |  | Nuclear Respiratory Factor 1 | | |
| PBX3 |  | PBX Homeobox 3 | | |
| PCBP1 |  | Poly(RC) Binding Protein 1 | | |
| PCBP2 |  | Poly(RC) Binding Protein 2 | | |
| PHF8 |  | PHD Finger Protein 8 | | |
| POLR2A |  | RNA Polymerase II Subunit A | | |
| POLR2G |  | RNA Polymerase II Subunit G | | |
| RAD21 |  | RAD21 Cohesin Complex Component | | |
| RAD51 |  | RAD51 Recombinase | | |
| RB1 |  | RB Transcriptional Corepressor 1 | | |
| RBBP5 |  | RB Binding Protein 5, Histone Lysine Methyltransferase Complex Subunit | | |
| RBFOX2 |  | RNA Binding Fox-1 Homolog 2 | | |
| RBM39 |  | RNA Binding Motif Protein 39 | | |
| RCOR1 |  | REST Corepressor 1 | | |
| RELB |  | RELB Proto-Oncogene, NF-KB Subunit | | |
| REST |  | RE1 Silencing Transcription Factor | | |
| RFX5 |  | Regulatory Factor X5 | | |
| RNF2 |  | Ring Finger Protein 2 | | |
| RUNX3 |  | RUNX Family Transcription Factor 3 | | |
| RXRA |  | Retinoid X Receptor Alpha | | |
| SIN3A |  | SIN3 Transcription Regulator Family Member A | | |
| SKI |  | SKI Proto-Oncogene | | |
| SMARCA4 |  | SWI/SNF Related, Matrix Associated, Actin Dependent Regulator of Chromatin, Subfamily A, Member 4 | | |
| SMARCA5 |  | SWI/SNF Related, Matrix Associated, Actin Dependent Regulator of Chromatin, Subfamily A, Member 5 | | |
| SMC3 |  | Structural Maintenance of Chromosomes 3 | | |
| SP1 |  | Sp1 Transcription Factor | | |
| SRF |  | Serum Response Factor | | |
| TAF1 |  | TATA-Box Binding Protein Associated Factor 1 | | |
| TAF15 |  | TATA-Box Binding Protein Associated Factor 15 | | |
| TAF7 |  | TATA-Box Binding Protein Associated Factor 7 | | |
| TBL1XR1 |  | TBL1X Receptor 1 | | |
| TBP |  | TATA-Box Binding Protein | | |
| TCF12 |  | Transcription Factor 12 | | |
| TFAP4 |  | Transcription Factor AP-4 | | |
| TRIM22 |  | Tripartite Motif Containing 22 | | |
| U2AF1 |  | U2 Small Nuclear RNA Auxiliary Factor 1 | | |
| USF1 |  | Upstream Transcription Factor 1 | | |
| USF2 |  | Upstream Transcription Factor 2, C-Fos Interacting | | |
| XRCC5 |  | X-Ray Repair Cross Complementing 5 | | |
| YY1 |  | YY1 Transcription Factor | | |
| ZBTB33 |  | Zinc Finger and BTB Domain Containing 33 | | |
| ZBTB7A |  | Zinc Finger and BTB Domain Containing 7A | | |
| ZFX |  | Zinc Finger Protein X-Linked | | |
| ZHX2 |  | Zinc Fingers and Homeoboxes 2 | | |
| ZNF143 |  | Zinc Finger Protein 143 | | |
| ZNF207 |  | Zinc Finger Protein 207 | | |
| ZNF217 |  | Zinc Finger Protein 217 | | |
| ZNF282 |  | Zinc Finger Protein 282 | | |
| ZNF384 |  | Zinc Finger Protein 384 | | |
| ZNF687 |  | Zinc Finger Protein 687 | | |
| ZSCAN29 |  | Zinc Finger and SCAN Domain Containing 29 | | |

Data shows ChIP-seq clusters (340 factors, 129 cell types from the ENCODE 3 database) for proteins which overlap with or are within the 1,768 bp region of the *INSR* promoter containing CpG islands (hg38/Chr19:7,293,330-7,295,097) as available in June 2020.
